# Biogeochemical and Ecomorphological Niche Segregation of Mediterranean Woody Species along a Local Gradient

**DOI:** 10.3389/fpls.2017.01242

**Published:** 2017-07-19

**Authors:** Enrique G. de la Riva, Teodoro Marañón, Cyrille Violle, Rafael Villar, Ignacio M. Pérez-Ramos

**Affiliations:** ^1^Estación Biológica de Doñana, Consejo Superior de Investigaciones Científicas Seville, Spain; ^2^Área de Ecología, Facultad de Ciencias, Universidad de Córdoba Córdoba, Spain; ^3^Instituto de Recursos Naturales y Agrobiología de Sevilla, Consejo Superior de Investigaciones Científicas Seville, Spain; ^4^CEFE UMR 5175, CNRS – Université de Montpellier – Université Paul-Valéry Montpellier – EPHE Montpellier, France

**Keywords:** functional ecology, growth forms, hypervolume, carbon isotope ratio, LDMC, leaf habit, nutrients, SLA

## Abstract

According with niche theory the species are specialized in different ecological niches, being able to coexist as result of a differential use of resources. In this context, the biogeochemical niche hypothesis proposes that species have an optimal elemental composition which results from the link between the chemical and morphological traits for the optimum plant functioning. Thus, and attending to the limiting similarity concept, different elemental composition and plant structure among co-occurring species may reduce competition, promoting different functional niches. Different functional habits associated with leaf life-span or growth forms are associated with different strategies for resource uptake, which could promote niche partitioning. In the present study, based on the biogeochemical niche concept and the use of resources in different proportions, we have focused on leaf traits (morphological and chemical) associated with resource uptake, and explored the niche partitioning among functional habits: leaf life-span (deciduous, evergreen, and semideciduous) and growth (tree, shrub, and arborescent-shrub). To this end, we have quantified the hypervolume of the leaf functional trait space (both structure and chemical composition) in a sample of 45 Mediterranean woody species from Sierra Morena Mountains (Spain) growing along a local soil resource gradient. Our results show consistent variation in functional space for woody communities distributed along the environmental gradient. Thus, communities dominated by deciduous trees with faster growth and a predominant acquisitive strategy were characteristic of bottom forests and showed highest leaf biogeochemical space. While semideciduous shrubs and evergreen (arborescent, trees) species, characterized by a conservative strategy, dominated ridge forests and showed smaller functional space. In addition, within each topographical zone or environment type, the foliar biogeochemical niche partitioning would underlie the species ability to coexist by diverging on leaf nutrient composition and resource uptake. Lower niche overlap among functional habits were found, which support that different growth forms and leaf life-habits may facilitate the coexistence of the woody species and niche partitioning along and within the gradient.

## Introduction

According to niche theory, species coexistence is promoted by ecological niche differences (MacArthur and Levins, 1967). While this idea is pivotal in community ecology, empirical evidences are contrasted for several reasons. First, theoretically the niche concept is a long and still-debated concept. Second, operationally, quantifying the whole niche of a species remains elusive. As an alternative, it has been proposed to replace niche axes by phenotypic trait axes in community ecology (McGill’s et al., 2006; Violle and Jiang, 2009). In plants, a myriad of studies has focused on plant morphological traits to analyze species niche partitioning along environmental gradients as well as niche partitioning within communities (e.g., Mason et al., 2011; Maire et al., 2012; Lamanna et al., 2014). However, the interpretation of these studies is often ambiguous given that a given morphological trait can be involved in several, sometimes opposed, assembly processes (de la Riva et al., 2017). Community species composition can be viewed as the result of a selection process named filtering, which combines species interactions (biotic filtering) and environmental adversity (abiotic filtering) operating simultaneously and favoring a trait convergence (McGill’s et al., 2006). At the same time, the process of niche partitioning tends to favor coexistence of species with divergent traits and complementarity in the use of resources (Maire et al., 2012). Therefore, stabilizing niche differences and relative fitness differences are determined by abiotic and biotic environment (HilleRisLambers et al., 2012). In this regard, given that plants all use the same types of resources (light, nutrients, CO_2_, water), the biochemical and ecomorphological characterization of plant niches may be a way to more accurately discriminate plants’ ecological strategies and niche partitioning along environmental gradients

Any plant to survive must occupy an environment with conditions that they can tolerate (Winemiller et al., 2015). Thus, habitat adaptation is determined primarily by abiotic constraints, which shape the species distribution along variable resource scenarios, and therefore, spatial trait segregation provides a starting point for building a niche scheme and performance measurements to predict how species respond to resource gradients (Winemiller et al., 2015). However, despite this spatial niche partitioning, species coexist within the same environment, because niche partitioning might involve some combination of strategies for resource use. In this context, a proposed mechanism of plant coexistence is based on the ‘biogeochemical niche’ hypothesis (Peñuelas et al., 2008). According to this hypothesis, species have an optimal elemental composition resulting from the link between the chemical composition and morphological traits for an optimum plant functioning (Peñuelas et al., 2008). Thus, differences in elemental composition and morpho-structure among co-occurring species may reduce competition, promoting different functional niche (Sardans et al., 2015). Within a determined environment type, the co-occurring species compete in the same space by different resources (not only nutrients, also by water and light) with different intensity, allowing the species coexistence in the same biome.

A great challenge is to identify groups associated with functional traits that allow for successful trait syndromes and ecological predictions. To this end, the assimilation and conservation of resources is a fundamental dimension. Wright et al. (2004) proposed a scheme, known as the ‘leaf economics spectrum,’ that assumes species segregation along environmental resources gradient, according with a plane of trait variation. Hence, this scheme runs from species with a conservative resource-use strategy (i.e., plants of slow growth, with tissues of high-density and low nutrient content) which are more abundant in low-productivity sites, to species from resource-rich sites with the opposite suite of traits, and associated to rapid resource capture (Wright et al., 2001; Villar et al., 2006; Poorter and Garnier, 2007). In this regard, different functional habits associated with leaf life-span (i.e., evergreen or deciduous) or growth forms (i.e., trees or shrubs) are associated with different strategies for resource uptake, which could promote niche partitioning (Mamolos et al., 1995; Niinemets and Kull, 2003; Lusk and Warton, 2007; Sardans et al., 2015; de la Riva et al., 2016a). Nevertheless, there is a large gap in our understanding on the influence of plant traits (leaf morphology and chemistry), leaf habit and growth forms as drivers of species niche partitioning (Sterck et al., 2011).

A variety of multivariate statistical methods have been developed to understand niche partitioning (e.g., Winemiller et al., 2015). However, more powerful tests of niche theories need to move beyond approaches based on species occurrence and instead to focus explicitly on trait-based approaches, recasting the theories in terms of functional space and diversity. The n-dimensional niche space model (Blonder et al., 2014), which is based on the Hutchinson’s multidimensional niche concept (Hutchinson, 1957), allows to quantify niche spaces by assessing the functional trait space that characterize the phenotypic volume occupied by a set of species (Violle and Jiang, 2009; Lamanna et al., 2014). A set of n variables, which represent key and independent biologically axes, are used to create a n-dimensional space, which is defined as “hypervolume.” The main advantage of this method is that the set of points are projected into a hyperspace, defining a high-dimensional shape which may include holes or complex geometrical features. In addition, this method allows quantifying the proportion of the hypervolumes that overlap in different habitats, i.e., the fraction of them sharing the same functional space (Blonder et al., 2014).

In a previous work, carried out at regional scale and at a whole plant level (i.e., quantifying leaf, root, and stem traits), we demonstrated that soil water scarcity led to lower plant functional space and trait segregation (de la Riva et al., 2017). Thus, communities of woody Mediterranean plants from arid and semi-arid shrublands displayed smaller functional space, with a higher degree of overlap between them. That pattern would reflect the species cluster around adaptive peaks, which are defined by sets of trait combinations associated with a given set of environmental attributes (Winemiller et al., 2015). In the present study, based on the biogeochemical niche concept and the use of resources in different proportions, we have focused on leaf traits (morphological and chemical) associated with resource uptake, and explored the niche partitioning among functional habits (leaf life-span and growth form). We have quantified the functional space based on leaf functional traits in a set of 45 woody species from Sierra Morena Mountains (South Spain) growing along a broad gradient of soil resources. Assuming that differences in leaf nutrient composition and morpho-structure reflect different ecological strategies, associated with niche partitioning, and so permitting coexistence (McGroddy et al., 2004; Wright et al., 2004; Chen et al., 2011), we hypothesized that: (i) As a consequence of different plant adaptations to maximize their fitness under determined environmental conditions; we expect a segregation in the foliar chemical composition and morphology among the species along the explored soil resource gradient (mainly soil water availability); (ii) the major differences in leaf chemical and morphological traits are found among species with contrasting growth forms and leaf life-spans. This implies that coexisting species would share their niches by using different ranges and proportions of resources, assuming trade-offs in resource allocation.

## Materials and Methods

### Study Area and Sampling Design

The Mediterranean forests studied are located in Sierra Morena Mountains, in the south of Spain (Córdoba province). The area is characterized by a continental-Mediterranean climate with cold, wet winters and dry, warm summers. Mean annual temperature is 17.6°C (with maximum values in summer reaching 40°C) and mean annual precipitation is 536 mm (with a 3-month period in summer without rainfall; data from AEMET for the years 1971–2000^1^). Several shrub and arborescent species, such as *Cistus albidus* and *Quercus coccifera*, are abundant in drier soils, while broad-leaf deciduous trees, such as *Alnus glutinosa* and *Fraxinus angustifolia*, are dominant in moister soils (see Supplementary Table 1 for details of the 45 studied species). One cultivate species (*Cydonia oblonga*) has been included, because is naturalized in the study area and could potentially alter the biogeochemical niche of the coexistent species. Twelve sampling sites distributed over four different south-facing slopes were selected along a topographic gradient (from ridges to valley bottoms; see Supplementary Figure 1) with the aim of spanning a broad range of variations in soil resource availability, mainly of soil water (Supplementary Figures 2, 3 and de la Riva et al., 2016b). The species composition was recorded by measuring the cover of each woody species intercepted by four 20-m transects in each sampling site.

### Trait Measurements

For trait measurements, we selected all the species appearing in the sampling transects, excluding only those with a relative abundance below 1% (in these cases it was difficult to find at least six individuals per species in the sampling site). This gave a total of 45 selected species, many of them appearing in more than one sampling site (Supplementary Table 1). We measured different leaf traits associated with resource uptake and conservation: two key functional traits related to morphology and associated with light capture and growth rate [specific leaf area (SLA)] and stress tolerance [leaf dry matter content (LDMC)], all the macronutrients (N, C, P, K, S, Ca, and Mg) and the isotopic ratio of carbon, which is related with gas exchange and water-use efficiency (Pérez-Harguindeguy et al., 2013).

Six samples of leaves from different individuals per species and site were collected in spring. Specific leaf area (leaf area per unit dry leaf mass; m^2^ kg^-1^) and LDMC (dry mass per unit of water-saturated fresh mass; g g^-1^) were measured according to the methods recommended by Pérez-Harguindeguy et al. (2013). Chemical composition was determined for a mixture of leaves, from six different individuals per species and sampling site (except N and C which were measured in each individual). N and C concentrations were measured using an elemental analyser (Eurovector EA, 3000; EuroVector SpA, Milan, Italy). The macronutrients P, K, S, Ca, and Mg were extracted by wet oxidation with concentrated HNO_3_ under pressure in a microwave digester, and analyzed by ICP-OES (Domínguez et al., 2012). Carbon isotope ratio (δ^13^C; ‰) was measured by combustion at 1020°C using a continuous flow isotope-ratio mass spectrometry system by means of Flash HT *Plus* elemental analyzer coupled to a Delta-V Advantage isotope ratio mass spectrometer via a CONFLO IV interface (Thermo Fisher Scientific, Bremen, Germany). The analytical measurement errors were ±0.1‰ for δ^13^C.

### Data Analysis

For all the statistical analyses, plant species were sorted into different groups according to (I) their topographical position (environment): Ridge Forest (hereafter ***RF***), Middle-slope Forest (hereafter ***MF***) and Riparian Forest (hereafter ***RiF***); (II) leaf habit: Evergreen (hereafter ***Ev***), Deciduous (hereafter ***De***) and Semideciduous (hereafter ***Sd***); and (III) growth form: Shrubs (hereafter ***Sr***), Trees (hereafter ***T***), Arborescent-Shrubs (hereafter ***ST***), and Climber (***C***) sensu lato. Semideciduous species are considered those that have the capacity to drop their leaves under severe drought conditions (Zunzunegui et al., 2005; Ciccarelli et al., 2016), and therefore they have functional differences with respect evergreens.

A general principal components analysis (PCA) was performed with the whole set of leaf structural and chemical traits (10 leaf variables) for the 100 observations (measurements) made of woody plants (of 45 species, as some species appear in more than one sampling site), to study the degree of leaf trait variation among them. We used linear mixed models (considering species as a random variable) to assess whether the PCA scores of the first, second and third components (see details in Supplementary Table 2) differed among all possible combinations of the following fixed explanatory factors: environment, leaf habit, and growth form.

The total niche space of the community was calculated by the estimation of the n-dimensional hypervolume (Blonder et al., 2014), from the trait space occupied by the total species group in the different categories (environment, leaf habit, and growth form; the number of observations for climbers was not enough to calculate the hypervolume). In order to reduce the number of dimensions (which is recommended for this analysis), we used the first three PCA axes to calculate the hypervolume for each group, using a multidimensional kernel density estimation (KDE) procedure (see Blonder et al., 2014 for mathematical details). The units of the hypervolumes are reported as the standard deviations of centered and scaled log-transformed trait values, raised to the power of the number of trait dimensions used (sd^number^ of dimensions). We also calculated the overlap between the hypervolumes of each group with the correlation analysis of the “hypervolume” package, which compares the similarity between different hypervolumes using the Sørensen index (see Blonder et al., 2015). A rarefaction analysis was performed to control for the effects of species richness on the hypervolume. Thus, for each environment type, leaf habit and growth form, we built 100 randomized communities composed of species drawn (nine observations) from the species pool of that group. Then, we calculated the hypervolume of each sample and performed a one-way ANOVA to compare the hypervolumes of the groups, independently of species richness. In addition, to assess the functional trait overlap within and among groups (leaf habit and growth form) within each environment type, we calculated the hypervolumes and the Sørensen index between each pair of the most representative groups along the gradient.

All these analyses were conducted in the R 2⋅10⋅0 statistical platform (R Development Core Team, 2011), using the packages “vegan” (Oksanen et al., 2013), “nlme” (Pinheiro et al., 2015), and “hypervolume” (Blonder et al., 2014).

## Results

### Species Variation

**FIGURE 1:**
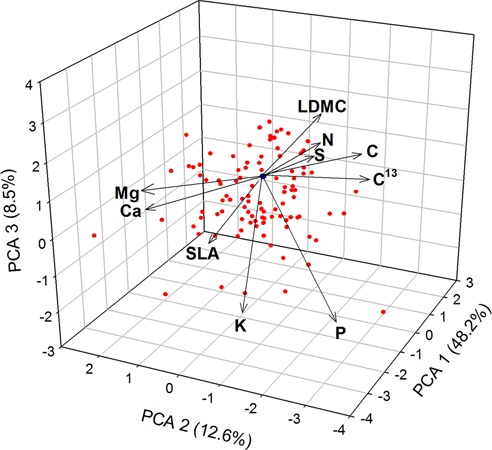
Principal components analysis (PCA) for the 10 leaf traits – morphological (LDMC and SLA), macronutrients (C, N, P, K, Mg, Ca, S) and carbon isotope ratio (C^13^) – and 45 woody species.

The overall PCA showed a clear separation among the 45 woody species in the volume defined by PCA_1_ (accounting for 48.2% variance), PCA_2_ (12.6% variance), and PCA_3_ (8.5% variance) (**Figure 1**). The PCA_1_ mostly reflected a gradient of increasing LDMC and C, and decreasing SLA, K and Mg; the PCA_2_ was mostly related in one extreme (negative values) with high N, P, and S and at the opposite extreme (positive values) with high values of Ca and Mg; the PCA_3_ was represented mainly by variations in N, S, P, and K (see variables scores in Supplementary Table 2).

**FIGURE 2:**
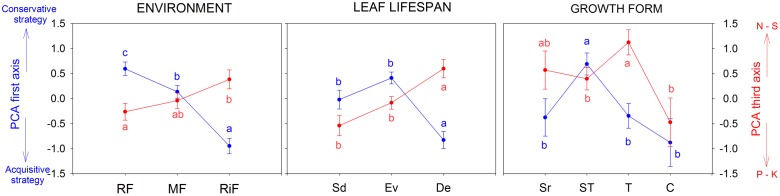
Differences among the scores of the first (leaf economic spectrum) and third (macronutrients) PCA axes, for species grouped according with environment (RF-Ridge forest, MF-Medium-slope, and RiF-Riparian forest), leaf life-span (Sd, semideciduous; Ev, evergreen; and De, deciduous) and growth forms (Sr, shrubs; ST, arborescent-shrubs; T, trees; and C, climbers). All the results were significant; Linear mixed models, *P* < 0.05 (Different letters means significant differences).

The woody species inhabiting the ridge forest (***RF***) had different trait values (well-separated according the first and the third PCA axes) from those living at the Riparian forest (***RiF***) (**Figure 2**). There was also a separation in the trait PCA between species grouped by leaf habit: Deciduous (***De***) were different from ***Sd*** and ***Ev***. With regards to growth form, there were differences among the arborescent shrubs (for PCA_1_), and trees (for PCA_3_) with arborescent shrubs and climbers (**Figure 2**); while no significant differences were found for PCA_2_.

### Functional Space

**FIGURE 3:**
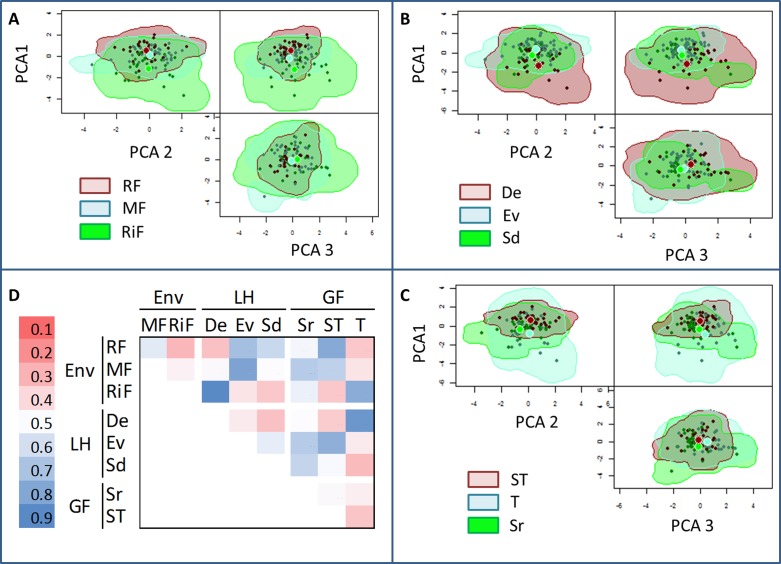
Estimated three-dimensional hypervolumes for: **(A)** Environment (RF, Ridge forest; MF, Medium-slope; and RiF, Riparian forest), **(B)** Leaf life-span (Sd, semideciduous; Ev, evergreen; and De, deciduous), **(C)** Growth forms (Sr, shrubs; ST, arborescent-shrubs; T, trees). **(D)** Sørensen similarity index among hypervolumes. Each plant dimension was based in each of the first three PCA axes (**Figure 1**).

**FIGURE 4:**
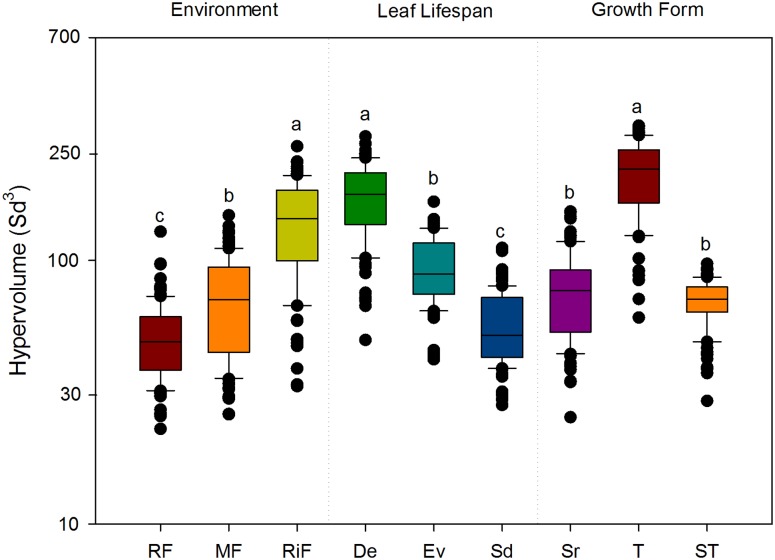
Size of the hypervolume variations for each environment, leaf life-span and growth form. Different letters indicate significant differences (*P* < 0.05), according to *post hoc* multiple pairwise comparisons (Tukey’s test). The ANOVA results were significant in all cases (*P* < 0.001).

The results from the *n*-dimensional hypervolume approach showed that the functional space was greatest for the riparian communities from riparian forest (**Figure 3A**), the group of deciduous species (**Figure 3B**), and the growth form “trees” (**Figure 3C**), in the three plant dimensions (PCA axes 1, 2, and 3). In addition, after standardizing for species richness, the functional space showed significant variation along the topographic gradient: the hypervolume was significantly greater at the riparian forest (*P* < 0.001; **Figure 4**). Semideciduous species showed the lowest hypervolume, while deciduous species had the highest. Among the growth form types, trees had the highest hypervolume (**Figure 4**).

### Overlapping Niches

The degree of overlapping among the hypervolumes of the different communities was variable, ranging from 28 to 90% (**Figure 3D**). Attending to the environment, the overlap degree of the ridge forest ***RF*** was greater with middle ***MF*** than with Riparian forest ***RiF***. Semideciduous type showed the highest overlap with evergreen type, and the lowest overlap with the deciduous group. Among the growth form types, the lowest degree of overlapping was found among the hypervolumes of trees, and arborescent-shrubs. In general, the degree of hypervolume-overlapping among different growth forms was relatively low, which indicate a trait space occupation more variable for growth forms than for environment or leaf habit.

**FIGURE 5:**
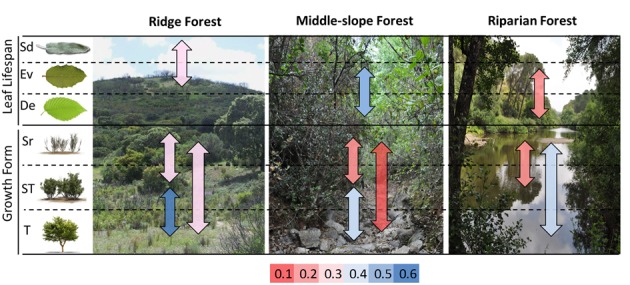
Sørensen similarity index for the functional niche space among species, grouped by leaf life-span and growth form, within the same habitat. Values are indicated by color according to the rank in the bottom of the figure.

Differences within the same groups were also remarkable (**Figure 5**). Only evergreen type was represented at the three environment types; it showed low functional niche space overlap (<0.3) with semideciduous (at the ridge) and (<0.2) with deciduous (at the riparian). The functional space was also very different among growth forms within the same environments (overlap < 0.4); with exception of trees and arborescent-shrubs in ridge forests, which had the highest overlap (**Figure 5**).

## Discussion

### Environment and Niche Partitioning

We have documented a consistent variation in functional space (based on leaf morphology and chemical composition) for woody communities distributed along the topographical gradient. Thus, communities dominated by deciduous trees with faster growth and a predominant acquisitive strategy (i.e., with high values of SLA and leaf nutrient concentration) were characteristic of riparian forests, while semideciduous shrubs and evergreen (arborescent, trees) species, characterized by a conservative strategy (i.e., with high-density leaves and a predominance of carbon-based structural defenses), dominated ridge forests. Hence, the first strong mechanism of species segregation and niche partitioning was related to habitat suitability. Along that local gradient of stress-productivity, plant establishment might be limited physiologically at one end (by stress tolerance) and by competition at the other end. Overall, leaf trait variation followed the patterns commonly associated to leaf economics spectrum (Grime, 1979; Wright et al., 2004).

This general niche differentiation began with the evolutionary accentuation of trait differences during the expansion of deciduous species on Cretaceous (Axelrod, 1966). The evolutionary and environmental drivers have determined this leaf chemical and morphological composition to improve plant functioning (Reich et al., 2003). However, we detected another trait dimension related with leaf concentration in some macronutrients (third PCA axis) that was not clearly aligned with the leaf economics spectrum. Other factors, like site differences or evolutionary history, might explain the lower values of leaf P and K associated to deciduous trees (Watanabe et al., 2007; Auger and Shipley, 2013). In a previous study on leaf chemical traits in 98 Mediterranean woody species, we also observed that leaf P and K were highly conditioned by the environment and phylogeny (de la Riva et al., accepted). These results suggest that leaf trait variation is not aligned along a single acquisition-conservation axis, and highlights the necessity of considering plant traits from other potentially independent leading dimensions such as those related with leaf chemical composition.

### Biogeochemical Niches

Within each topographical zone or environment type, the leaf biogeochemical niche partitioning would underlie the species ability to coexist by diverging on leaf nutrient composition and resource uptake. Thus, riparian communities from valley bottom showed the highest leaf biogeochemical space, which supports the existence of a greater number of functionally different species, as far as the acquisition of resources is concerned. In a regional study, the higher functional diversity (at whole plant level) was also found in wetter environments along the aridity gradient (de la Riva et al., 2017). On the contrary, the lowest diversity could be due to resource scarcity limiting the establishment of species that are not physiologically able to tolerate such abiotic constraints; as a consequence, the range of functional traits is reduced within the range of viable traits that allow plants to persist in that arid environment, in detriment of the functional space (de la Riva et al., 2016c). The more productive environments, however, are able to increase resource heterogeneity, and promote the coexistence of the species with different demand of them (de la Riva et al., 2017).

Niche partitioning within the same growth form or leaf life-span may also facilitate the coexistence among woody species. Riparian forests were dominated by deciduous tree species, which in fact was the life-span type with higher biogeochemical and morphological niche functional space. The dense shade created by the trees in this type of environment likely may induce a high among-species competition for light. However, woody species may also exhibit some degree of plasticity in nutrient uptake to respond to this competition. Plant species adapted to these productive environments, where nutrients have usually intermittent availability, might respond better to these temporal changes showing higher capacities for taking up resources and higher nutrient flexibility (Aerts, 1999; Sardans et al., 2015). Thus, our results suggest that differences in niche functional space (implying different strategies of resource capturing) among coexisting species would potentially allow to buffer competition in these resource-rich environments.

On the contrary, we detected smaller functional space in more stressful environments (i.e., ridge forests). This result could be explained because species growing in poor environments comprise traits which lead to nutrient retention and higher nutrient-use efficiency, which seem to confer a lower capacity to change their functioning in response to environmental changes (Aerts, 1999). In addition, plant size seems to be related with root expansion and nutrient uptake. Thus smaller species (more frequent in resource-poor environments) tend to show lower values of leaf nutrients than trees as a result of their lower capacity to maintain larger root systems and explore larger soil volumes to uptake nutrients (Niinemets and Kull, 2003 and references therein). As a consequence, shrub and arborescent-shrub species had lower niche space than trees. The combined effects of the slow population dynamics in poor environments (Wright, 2002), and their lower capacity to alter their elemental composition independently of the nutrient pulses (Sardans et al., 2015) would facilitate the coexistence of ecologically equivalent species.

### Growth Forms and Niche Partitioning

Differences in growth form and leaf life-span may facilitate the coexistence of woody species and their niche partitioning, as showed by the lower functional overlap among them. In spite of the general patterns found along the topographical gradient (e.g., dominance of deciduous trees at riparian forests, and evergreen arborescent-shrubs at ridge forests), strong divergence were also found between their functional spaces within the same habitat type. Thus, the low degree of overlap between the functional space of deciduous trees and evergreen arborescent-shrubs at the riparian forest reflects a niche partitioning, probably promoted by light competition and resource partitioning. The dense-shaded forest understory is a relatively stressful habitat for plants (in terms of light availability), which promotes a resource gradient also related with leaf economics spectrum (Lusk and Warton, 2007). On the one hand, deciduous trees (such as *Alnus glutinosa* and *Fraxinus angustifolia*) are effective competitors, since they are able to rapidly acquire nutrients and water, that allow them to grow faster and compete more efficiently for light interception (Lambers and Poorter, 1992; Wright et al., 2001); on the other hand, evergreen shrub species (such as *Rubus ulmifolius*) had higher LMA, lower nutrient concentration, and are associated with shade tolerance (Williams et al., 1989; Lusk, 2002).

In the opposite extreme of the environmental gradient – at the ridge forests – woody species with semideciduous life-span seem to display more acquisitive patterns than evergreens. Shorter leaf life-span is related to higher relative nutrient requirement and lower resistance to physical hazards (Ryser, 1996; Pérez-Harguindeguy et al., 2013). In this sense, the habit of semideciduous leaf could be considered a facultative strategy, typical of shrubs in dry Mediterranean conditions (Axelrod, 1966; Zunzunegui et al., 2005). That strategy allows them to be effective competitors during favorable conditions, while they shed partly or completely their leaves during summer, so reducing water loss by transpiration (Zunzunegui et al., 2005; Ciccarelli et al., 2016; Bongers et al., 2017). Therefore, the shrub species might be segregated along the resource gradient through a trade-off between growth rate and survival. In summary, changes in plant structure such as growth form or leaf life-span, might reduce competition by differences in the ability to capture and use resources, supporting that limiting similarity was operating within the communities (Stubbs and Wilson, 2004).

## Author Contributions

EdlR designed the study, harvested samples, collected the data, analyzed the data, wrote the first draft, and prepared the manuscript. TM, CV, RV, and IP-R designed the study and prepared the manuscript.

## Conflict of Interest Statement

The authors declare that the research was conducted in the absence of any commercial or financial relationships that could be construed as a potential conflict of interest.

## Footnotes

1 http://www.aemet.es/es/serviciosclimaticos/datosclimatologicos
