# Supplementary material for: Biogeochemical and Ecomorphological Niche Segregation of Mediterranean Woody Species along a Local Gradient
Source: Front Plant Sci. 2017 Jul 19;8:1242. doi: 10.3389/fpls.2017.01242 (PMC5515866; doi:10.3389/fpls.2017.01242)
Supplement: Supplementary file 1 [file Data_Sheet_1.docx]

Supplementary Material

Article Title: Biogeochemical and ecomorphological niche segregation of Mediterranean woody species along a local gradient

Corresponding Author: [enga70@gmail.com](mailto:enga70@gmail.com)

**Supplementary Table 1.** Species studied, growth form (Shrubs, ***Sr***; Trees, ***T***; Arborescent-Shrubs, ***ST***; Climber, ***C***), leaf habit (Evergreen, ***Ev***; Deciduous, ***De***; and Semideciduous, ***Sd***) and presence in the different sampling sites (Ridge Forest, ***RF***; Middle-slope Forest, ***MF***; Riparian Forest, ***RiF***).

**Supplementary Table 2.** Results of the principal components analysis (PCA) for leaf nutrients and morphology traits. For each axis, the eigenvalues and proportion of variance explained are provided. Values in bold means that the variable is strongly related to the PCA axis

**Supplementary Figure 1.** Schematic overview of the location of twelve sampling sites distributed over four different south-facing slopes along a topographic gradient (from ridges to valley bottoms). The species composition was recorded by measuring the cover of each woody species intercepted by four 20-m transects in each sampling site. Abbreviations: Virgen de Linares, **VL**; Baños de Popea, **P**; Las Tonadas, **VV**; Cardeña, **C**; Ridge Forest, **RF**; Middle-slope Forest, **MF**; Riparian Forest, **RiF**.


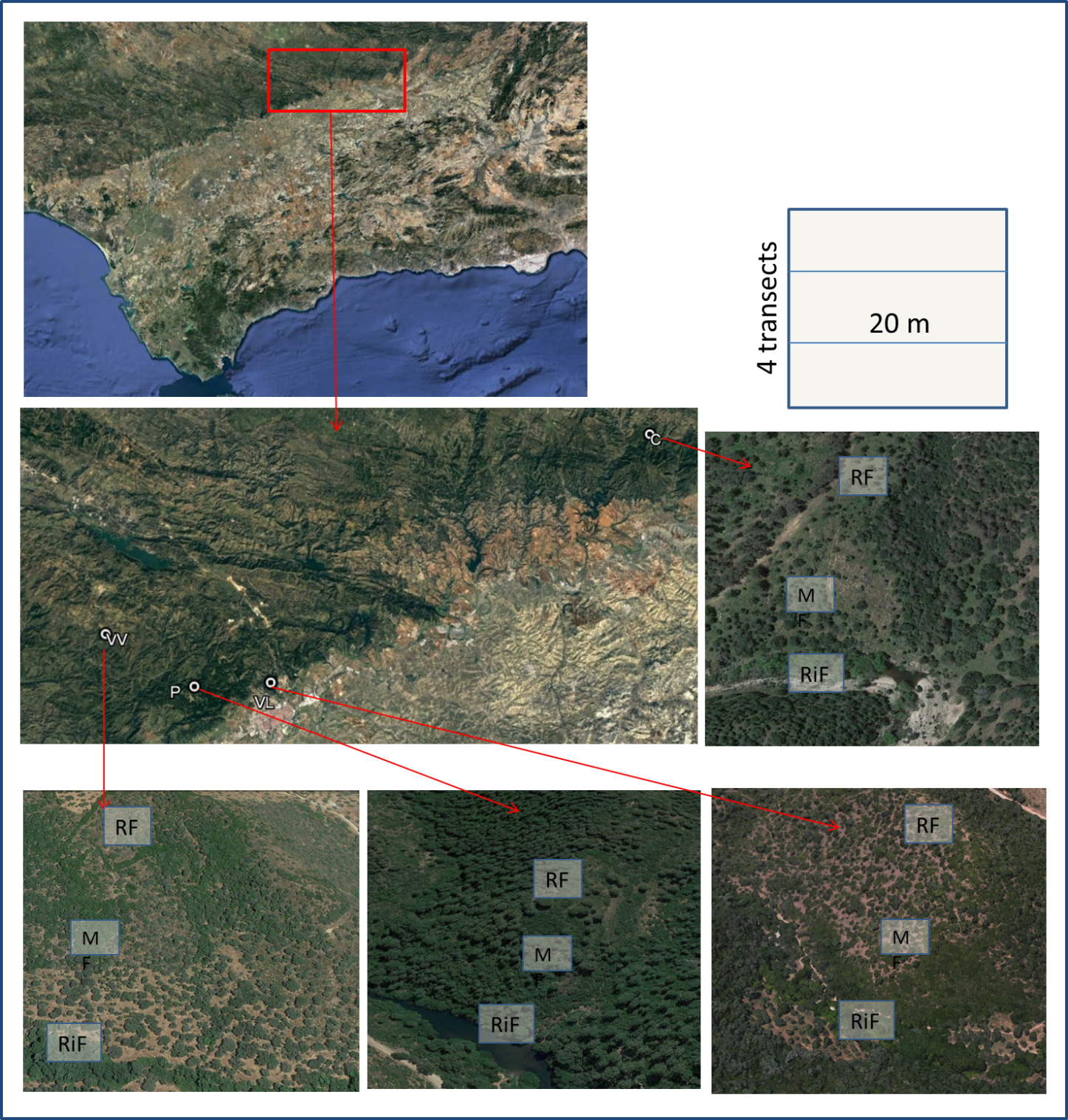


**Supplementary Figure 2.** Results of a one-way ANOVA between topographical zones and soil water storage (*P*< 0.05). Vertical bars denote 0.95 confidence intervals. Abbreviations: Ridge Forest (**RF**), Middle-slope Forest (**MF**) and Riparian Forest (**RiF**). See Note *.


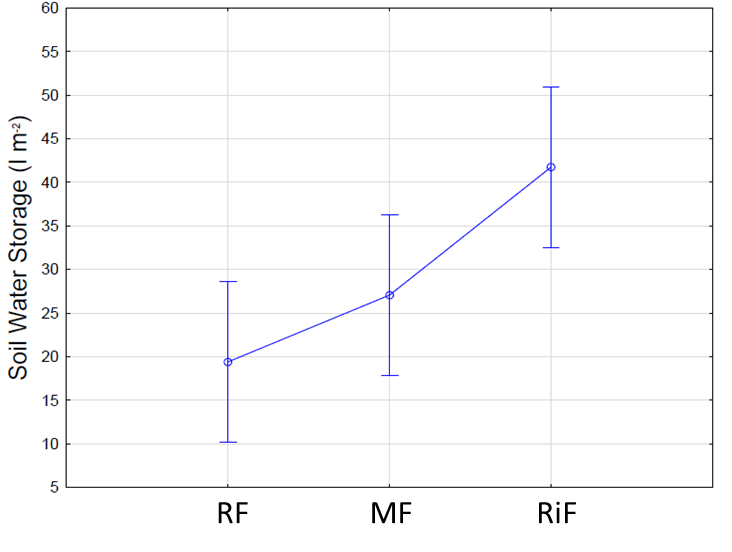


**Supplementary Figure 3**. Relationships between the first PC axis (leaf morphology and nutrients) and Soil Water Storage (data from de la Riva et al. 2016b). The R^2^ from linear regression and P value of the linear mixed model are shown. See Note *.


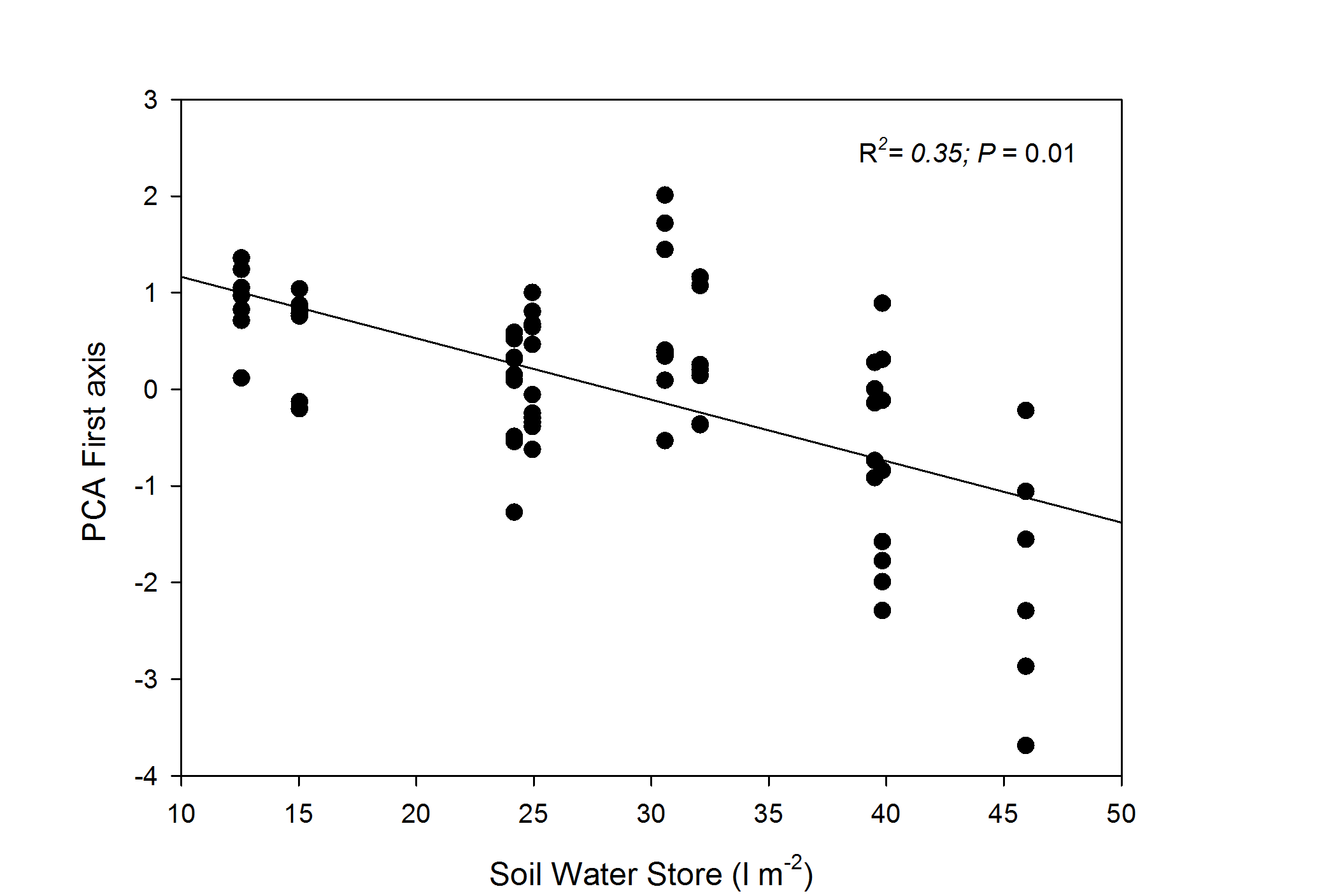


***Note**** Data of Soil Water Storage from de la Riva et al. (2016b). The sampling sites of Cardeña have been deleting from these analysis (soil water storage was not measured in these sites).
